# MiCellAnnGELo: annotate microscopy time series of complex cell surfaces with 3D virtual reality

**DOI:** 10.1093/bioinformatics/btad013

**Published:** 2023-01-11

**Authors:** Adam Platt, E Josiah Lutton, Edward Offord, Till Bretschneider

**Affiliations:** Department of Computer Science, University of Warwick, Coventry CV4 7AL, UK; Department of Computer Science, University of Warwick, Coventry CV4 7AL, UK; Department of Computer Science, University of Warwick, Coventry CV4 7AL, UK; Department of Computer Science, University of Warwick, Coventry CV4 7AL, UK

## Abstract

**Summary:**

Advances in 3D live cell microscopy are enabling high-resolution capture of previously unobserved processes. Unleashing the power of modern machine learning methods to fully benefit from these technologies is, however, frustrated by the difficulty of manually annotating 3D training data. MiCellAnnGELo virtual reality software offers an immersive environment for viewing and interacting with 4D microscopy data, including efficient tools for annotation. We present tools for labelling cell surfaces with a wide range of applications, including cell motility, endocytosis and transmembrane signalling.

**Availability and implementation:**

MiCellAnnGELo employs the cross-platform (Mac/Unix/Windows) Unity game engine and is available under the MIT licence at https://github.com/CellDynamics/MiCellAnnGELo.git, together with sample data. MiCellAnnGELo can be run in desktop mode on a 2D screen or in 3D using a standard VR headset with a compatible GPU.

**Supplementary information:**

Supplementary data are available at *Bioinformatics* online.

## 1 Introduction

In recent years, artificial intelligence (AI) has become a powerful tool for bioimage analysis. A major roadblock for the application of AI is obtaining manually annotated training data, which requires human experts to label image features on a computer screen, a tedious process prone to a high error rate. Standard approaches for labelling 3D objects use 2D cross-sections to view and annotate the volumes, which, because single 3D objects can appear disjointed in 2D sections, require excessive input from the annotator, limiting the number of annotations produced. We aim to address this issue with our software, MiCellAnnGELo (*Mi*croscopy and *Cell Ann*otation *G*raphical *E*xperience and *L*abelling to*o*l), an easy-to-use virtual reality (VR) interface for annotating dynamic biological surfaces.

The use of VR for data visualization dates back over 25 years (e.g. Frühauf and Dai, 1996). Recent advances in the graphics processing unit (GPU) and VR headset technology have greatly expanded the range of applications of this technology. ConfocalVR (Stefani *et al.*, 2018), Arivis VisionVR (Conrad *et al.*, 2020) and syGlass (Pidhorskyi *et al.*, 2018) are examples of VR software for annotating 3D biological image volumes. SlicerVR (Pinter *et al.*, 2020) is a VR visualization plugin for the open-source software 3D Slicer (Fedorov *et al.*, 2012). TeraVR (Wang *et al.*, 2019) provides a specialized VR application for neuron tracing in 3D image volumes, with functionality for placing markers and surface visualization, as part of the open-source software Vaa3D (Peng *et al.*, 2010). ChimeraX (Pettersen *et al.*, 2021) is primarily focused on molecular visualization and analysis but has also been applied to biological image analysis and surface visualization (Driscoll *et al.*, 2019; Quinn *et al.*, 2021).

MiCellAnnGELo aims to facilitate fast annotation of 3D microscopy movies. We have focused development on the annotation of time series of cell surfaces, which is a less computationally intensive task than directly interacting with the 3D microscopy movies, allowing annotation of large movies without requiring high GPU specifications. This is made possible by recent advances in cell segmentation methods (e.g. Arbelle *et al.*, 2022; Eschweiler *et al.*, 2022; Lutton *et al.*, 2021) allowing the extraction of the cell surface meshes. The example meshes used in the following additionally contained fluorescence data, taken from the source image using local maximum fluorescence (see Lutton *et al.*, 2022, for details). Two annotation methods are available in MiCellAnnGELo: a ‘mesh painting’ feature that allows fast labelling of the surfaces for a whole time series, while markers can be placed for feature tracking. We are releasing the software as an open-source tool to facilitate use and development within the community. Finally, MiCellAnnGELo is designed for VR visualization, allowing development to focus on streamlining this environment for annotation.

## 2 Software features

MiCellAnnGELo is a cross-platform software program that provides an immersive environment for the rapid annotation of a series of triangulated surface meshes. Meshes with single- or dual-channel colour mappings can be displayed and annotated in the environment. The software provides both VR and desktop interfaces, with easy interchange between these modes.

**Fig. 1.**
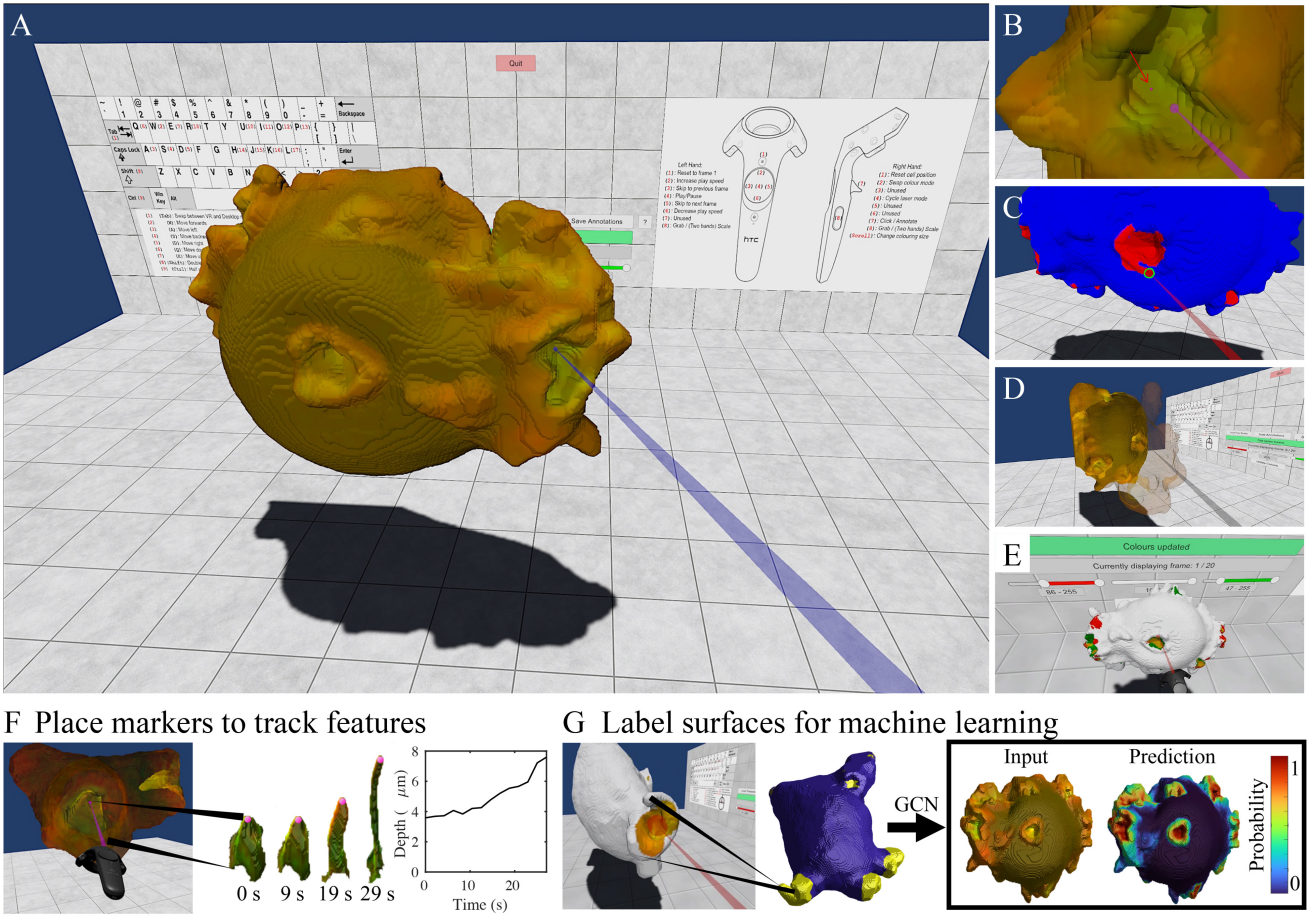
(**A**) Overview of the MiCellAnnGELo environment. (**B**) Placement of a marker (arrow) using the laser pointer. (**C**) Mesh painting in the two-tone annotation mode using the size-adjustable paint tool (circled region). (**D**) Transparency of part of the surface can be adjusted using the laser pointer. (**E**) Identifying colour-dependent features can be facilitated through colour thresholding. (**F**) One application of the software is the ability to rapidly place markers (left) in a sequence of surfaces, allowing manual tracking of surface features (middle), which can be paired with a feature detection method to allow time-dependent measurements to be made (right). (**G**) A second application of the software is to generate training data by painting labels on the surface (left), which can be used by a machine learning algorithm, e.g. a graph convolutional neural network (GCN) to predict features from input labels (right panel)

The user interface is designed with simplicity in mind, allowing the user to load, explore, and annotate data with ease. As can be seen in Figure 1A, the environment consists of the surface mesh itself and a wall-mounted user interface, which provides controls for loading and saving data, and for adjusting surface colour and opacity. Additionally, controller layouts for both VR and desktop modes are displayed on the wall for ease of use. In both VR and desktop environments, functions including changing annotation modes or surface representations, moving forwards/backwards in time, and playing/pausing the series are mapped to single buttons, enabling rapid multi-modal annotation of a sequence.

Annotations can be made by placing markers (Fig. 1B, Supplementary Video S1) and by mesh painting (Fig. 1C, Supplementary Video S2). Marker placement allows features to be marked with a single shot per frame, allowing rapid manual tracking of surface features across multiple frames. Painting allows rapid labelling of larger structures on the surface, with an easily adjustable brush size and eraser mode. The painted surface can be visualized either as a two-tone image or as a cut-out image with mesh colours being blocked out in unlabelled areas (Fig. 1G left, Supplementary Video S2).

A number of view controls have been added to enable easier labelling of data. In VR mode, the user can freely adjust the surface mesh position and size (Supplementary Video S3), allowing the user to rapidly change perspectives. For annotating more complex surfaces, the user can reduce the opacity of parts of the surface (Fig. 1D, Supplementary Video S4). Finally, the mesh colours can be adjusted using controls on the wall UI (Fig. 1E, Supplementary Video S4), allowing increased visibility of colour-dependent features.

Surface meshes can be loaded into the environment from a sequence of .ply files. This format stores colour data and can be generated from surfaces in many image analysis software applications [e.g. python via PyVista (Sullivan and Kaszynski, 2019) and Matlab via plywrite: https://www.mathworks.com/matlabcentral/fileexchange/55171-plywrite]. MiCellAnnGELo takes the first two channels of the .ply files as colour data. Marker annotations are exported as .csv files encoding the frame number, spatial coordinates and vertex index in the surface mesh of each marker (example shown in Supplementary Video S1). Painted surfaces are exported as .ply files, keeping the original colours in the first two channels and placing the paint labels in the third channel and can be retrieved in future sessions by selecting this sequence in the load menu. These labels can be read [e.g. via PyVista (Sullivan and Kaszynski, 2019), Meshlab (Cignoni *et al.*, 2008), 3D Slicer (Fedorov *et al.*, 2012) and Matlab via plyread: https://www.mathworks.com/matlabcentral/fileexchange/47484-plyread-m] for further analysis in other software by extracting the blue channel.

We demonstrate two example use cases in Figure 1F–G. In both cases, annotations were made on the surface of a cell undergoing macropinocytosis (cell drinking), with the aim to identify the concave structures, referred as cups, that are associated with this process. In the first, we used the marker placement tool to mark the centres of individual cups over time. Applying a threshold to the green channel gives an approximate shape of the marked cup in each frame, allowing time-dependent geometric variation of the cups to be measured. Using the mesh painting tool to label the cups enables machine learning methods to be applied, yielding a more accurate representation of the cups. The labels were used as training data for a graph convolutional neural network, which could then isolate the cups in a much larger set of surfaces.

## 3 Conclusion

MiCellAnnGELo is a cross-platform open-source software application designed to allow rapid annotation of surface series using VR technology. The software is streamlined and easy to use, with a range of viewing and annotation options. In future developments, we aim to introduce functionality for 3D volumetric data and multi-object series and allow more input and output formats. We aim to develop this software as a community resource project, allowing further development to be geared towards providing a VR software solution for a wide range of microscopy use cases and easy integration into existing pipelines.

## Supplementary Material

btad013_Supplementary_Data

## Data Availability

sample data available at https://github.com/CellDynamics/MiCellAnnGELo.
